# ARC: a framework for access, reciprocity and conduct in psychedelic therapies

**DOI:** 10.3389/fpsyg.2023.1119115

**Published:** 2023-05-11

**Authors:** Meg J. Spriggs, Ashleigh Murphy-Beiner, Roberta Murphy, Julia Bornemann, Hannah Thurgur, Anne K. Schlag

**Affiliations:** ^1^Division of Psychiatry, Department of Brain Sciences, Centre for Psychedelic Research, Imperial College London, London, United Kingdom; ^2^Drug Science, London, United Kingdom; ^3^Department of Psychology, Royal Holloway University of London, London, United Kingdom; ^4^South West London and St George’s Mental Health NHS Trust, London, United Kingdom

**Keywords:** psychedelic assisted therapy (PAT), ethics, equity, co-design, psychotherapy

## Abstract

The field of psychedelic assisted therapy (PAT) is growing at an unprecedented pace. The immense pressures this places on those working in this burgeoning field have already begun to raise important questions about risk and responsibility. It is imperative that the development of an ethical and equitable infrastructure for psychedelic care is prioritized to support this rapid expansion of PAT in research and clinical settings. Here we present Access, Reciprocity and Conduct (ARC); a framework for a culturally informed ethical infrastructure for ARC in psychedelic therapies. These three parallel yet interdependent pillars of ARC provide the bedrock for a sustainable psychedelic infrastructure which prioritized equal access to PAT for those in need of mental health treatment (Access), promotes the safety of those delivering and receiving PAT in clinical contexts (Conduct), and respects the traditional and spiritual uses of psychedelic medicines which often precede their clinical use (Reciprocity). In the development of ARC, we are taking a novel dual-phase co-design approach. The first phase involves co-development of an ethics statement for each arm with stakeholders from research, industry, therapy, community, and indigenous settings. A second phase will further disseminate the statements for collaborative review to a wider audience from these different stakeholder communities within the psychedelic therapy field to invite feedback and further refinement. By presenting ARC at this early stage, we hope to draw upon the collective wisdom of the wider psychedelic community and inspire the open dialogue and collaboration upon which the process of co-design depends. We aim to offer a framework through which psychedelic researchers, therapists and other stakeholders, may begin tackling the complex ethical questions arising within their own organizations and individual practice of PAT.

## 1. Introduction

One in eight people in the world today are living with a mental health difficulty (World Health Organization, 2022), and there are increasing demands for the development of new approaches to mental health treatment. Despite an overall growth in mental health research, the proportion of studies looking at new interventions, particularly of a pharmacological nature, has declined, with many large pharmaceutical companies withdrawing funding. As a result, there have been few, if any, major pharmaceutical breakthroughs since the 1950s (Hyman, 2013; Stephan et al., 2016; Nutt et al., 2020; Wortzel et al., 2020). There has also been a growing recognition of the wider social, ecological, and socio-economic determinants of mental wellbeing and the health inequalities this represents. This has shaped the resulting calls for a greater focus on integrative, collaborative, and community-based care to better support mental wellbeing for all (Shim et al., 2014; Commission for Equality in Mental Health, 2020; Knifton and Inglis, 2020; World Health Organization, 2022).

Psychedelic-assisted therapy (PAT)^1^ has been suggested as a paradigm shift that could address many of the challenges the fields of psychiatry face (Schenberg, 2018; Nutt et al., 2020, Nutt et al., 2022; Petranker et al., 2020; Lu, 2021). Since 2006, there has been extensive growth, in the number of clinical trials conducted, the potential conditions for which PAT is being investigated, as well as in the number of research centers across the globe that are undertaking these trials (Aday et al., 2020; Reiff et al., 2020; Yaden et al., 2021). Where once funding came primarily from philanthropists, corporate enterprises and private investors are now interested in this new approach and are funding a rapid expansion of the field (Phelps et al., 2022; Schwarz-Plaschg, 2022).

Drawing upon the (thus far) positive results of research, a media spotlight has also been placed upon psychedelics (Pilecki et al., 2021; Williams M. L. et al., 2021). Yet, psychedelics remain illegal in most jurisdictions across the world. Given the success of grassroots movements in instigating psilocybin decriminalization initiatives in parts of the US, and recent regulatory changes permitting the prescription of psilocybin and MDMA in Australia from July 2023 onward, it is possible similar public interest initiatives will develop in other parts of the world. Public support has also facilitated early access and compassionate use schemes in some countries that bypass the slow and meticulous pace of research where some deem existing evidence as sufficient to claim it unethical to withhold the treatment for those in need (Greif and Šurkala, 2020). This growing awareness has come alongside rising rates of naturalistic psychedelic use and increasing numbers of psychedelic retreats being offered in many countries globally (Yockey et al., 2020; Killion et al., 2021; Yockey and King, 2021; Glynos et al., 2022). A greater demand for reliable harm reduction information is demonstrated by the growing number of training programmes, referral networks, and psychedelic integration circles run for and by healthcare professionals (Pilecki et al., 2021).

Standing at the helm of a potential paradigm shift, the psychedelic research community now has the opportunity to help steer the future of PAT in a fair and sustainable manner. Within the broader context, there is a pressing need to better understand how these treatments might be most equitably and ethically delivered. The speed at which the field of PAT is moving has already begun to unearth cracks. Reports of unethical conduct have surfaced from both inside and outside the legal framework [Peluso et al., 2020; Psymposia and New York Magazine Cover Story (Power Trip), 2022; Schwarz-Plaschg, 2022]. Additionally, the assimilation of psychedelics into a purely biomedical framework risks repeating historical injustices and exacerbating inequities (Devenot et al., 2022; Schwarz-Plaschg, 2022). This has led some to question the capacity of this budding field to maintain ethical integrity (Williams M. L. et al., 2021; Yaden et al., 2021; Phelps et al., 2022) and has resulted in a flourish in ethical comments in both the public and academic domain (Smith and Sisti, 2020; Brennan et al., 2021; Pilecki et al., 2021; Thal et al., 2021; Williams M. L. et al., 2021; McMillan, 2022; Smith and Appelbaum, 2022). Ethical and practice guidelines for how, when, and by whom PAT should be conducted have been developed by different actors including research establishments [e.g., MAPS (Carlin and Scheld, 2019)], professional bodies [e.g., Psychedelic Association of Canada (PAC, 2022)], and community led/grassroots organizations [e.g., the North Star Pledge (North Star Ethics Pledge, 2020) and Chacruna’s Indigenous Reciprocity Initiative (Chacruna, 2021)]. At present, this growing wisdom is diffuse and often at times context specific. With no overarching framework from which to work across sectors, there is a risk that many of these important ethical questions may fall to the wayside as the psychedelic movement accelerates forward.

**FIGURE 1:**
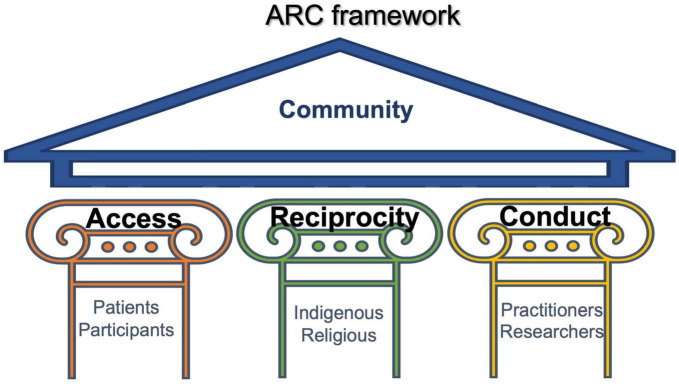
The ARC framework consists of three pillars: Access, Reciprocity and Conduct. The strength of each pillar calls upon specific actors, but it is their interdependence that is critical to an equitable and culturally informed ethical framework.

Here we present Access, Reciprocity and Conduct (ARC); a framework for ethically informed ARC in psychedelic therapies (Figure 1). The three pillars of ARC represent a commitment to equitable access to psychedelic therapies (Access), a respect for traditional and spiritual uses of psychedelics (Reciprocity), and the safe and ethical delivery of PAT in clinical settings (Conduct). Each pillar subsumes its own unique ethical challenges that are far-reaching and multifaceted. In an effort to balance complexity and parsimony, the ARC framework provides a scaffold for in-depth independent explorations of each pillar, while offering opportunity for shared learning. As depicted in Figure 1, different stakeholders hold particular relevance to each pillar, however, their interdependence is critical for supporting the continued growth of policy, industry, research, clinical and community-based infrastructure. Here, we provide background on each of these pillars, before outlining the development process for ARC.

## 2. ARC pillars

### 2.1. Access

It is well documented that mental health disparities exist at global, national, and local levels (Weich et al., 2001; Amaddeo and Jones, 2007; Evans-Lacko et al., 2018). The social determinants of mental health outcomes are also well established, showing that marginalized groups (those who experience social and political inequality) are disproportionately affected by mental distress (Alegría et al., 2018). Low socioeconomic status is related to both poor mental health outcomes and limitations in accessing care, with poverty being both a causal factor and a consequence of mental health difficulties (Alegría et al., 2018). Discrimination related to race, ethnicity, immigrant status, sexual orientation and other marginalized identities are also strongly associated with negative mental health outcomes (Benoit et al., 2015; Berger and Sarnyai, 2015; Lee et al., 2016; Hynie, 2018; Pachter et al., 2018).

There are multiple barriers to accessing mental healthcare for marginalized groups. There are not only practical barriers (e.g., distance, costs of transport, loss of income to attend appointments) but also psycho-social and cultural barriers (e.g., culturally inappropriate models of illness, stigma, racism and discrimination, history of abuses by mental healthcare and research, and a resulting fear and mistrust toward accessing healthcare services (Amaddeo and Jones, 2007; Memon et al., 2016). As such, even in countries where efforts are made to improve equal access through the national provision of free healthcare (e.g., through the UK National Health Service), the distribution of *needs-based* care is socially patterned, meaning that those from marginalised^2^ groups who do access mental healthcare, are still more likely to receive a poor service, and one which is not designed to adequately meet their needs (Watt, 2018).

Psychedelic assisted therapy runs the risk of also exacerbating these existing inequalities in access to healthcare. Misconceptions and laws surrounding drugs, including psychedelics, propagated by historical mistruths in the media and “the war on drugs” have disproportionately impacted marginalized groups by the disruption of communities, as well as racial stereotyping, profiling, and discrimination (Hutchison and Bressi, 2021; Rea and Wallace, 2021). Additionally, a history of exploitation in psychedelic research carried out between the 1950s and 1980s (Strauss et al., 2022) has left a legacy of mistrust toward science and healthcare among underrepresented communities. This has all played a part in perpetuating inadequate representation of marginalized groups within modern research settings; both in participants of clinical trials and, in some research teams themselves (Michaels et al., 2018, 2022; Buchanan, 2021; Williams M. T. et al., 2021). This under-representation means the results of PAT research may not be generalizable to marginalized groups. Consequently, this may form part of a vicious cycle in which some PAT practitioners and models do not adequately consider the necessary adaptations for working appropriately and sensitively with the people represented. If legalized, PAT may also be a prohibitively expensive treatment, meaning there is a serious need to consider affordable models of care across disparate healthcare contexts from the start. If equity is not prioritized in the current research context, or in the development and regulation of legally available treatments, the risk of perpetuating healthcare inequalities is high (Rea and Wallace, 2021).

Well-conducted research, which invites co-design and collaboration from members of marginalized groups, may contribute to shaping more widely applicable models of psychedelic care. However, this will not happen without deliberate and conscious action. The goal of the “Access” pillar of the ARC framework is to identify priority areas for PAT in research and clinical contexts to suggest actionable steps toward the development of inclusive, equitable and culturally sensitive models of care and practice.

### 2.2. Reciprocity

Many Indigenous peoples have stewarded psychedelics as traditional medicines for millennia, cultivating relationships with and accumulated knowledge on various plants, fungi and cacti, some of which are now used in PAT (Celidwen et al., 2023).

As plant psychedelics mature as medicines in Western contexts, a degree of commercialization of these compounds and their sacred ancient practices seems inevitable. Numerous companies and individuals are already profiting from speculative investments with few, if any, benefits accruing to Indigenous peoples (Williams et al., 2022).^3^ Rather, Indigenous peoples are often left out of the sector, as the field is currently widely represented by Westerners. This raises moral and ethical issues, such as those related to cultural appropriation, patenting of “the sacred” and exclusionary practices in research and praxis. These must be addressed if the psychedelic ecosystem is to develop in an equitable and sustainable manner.

Initiatives for addressing reciprocity have been launched by various organizations. For example, the Indigenous Reciprocity Initiative helps Indigenous peoples create conditions for medicine development (Chacruna, 2021), while other initiatives focus on avenues for financial support [e.g., Grow Medicine (2022) and the Indigenous Medicine Conservation Fund (2022)]. The variability of these approaches reflects the complexity of their aims and objectives, and these are yet to be standardized into guidelines for if and how to give back to traditional knowledge carriers of ancient plant medicines.

Recently, presenting an all-encompassing approach, an Indigenous-led globally represented group of practitioners, activists, scholars, lawyers, and human rights defenders convened to formulate a set of ethical guidelines concerning traditional Indigenous medicines current use in Western psychedelic research and practice (Celidwen et al., 2023). Appreciating the challenges in discussing reciprocity and benefit sharing, the group nevertheless identified eight interconnected ethical principles for engaging with Indigenous peoples in relation to psychedelic research and practise: Reverence, Respect, Responsibility, Relevance, Regulation, Reparation, Restoration, and Reconciliation. This transdisciplinary and transcultural group aims to continue their important work by further examining the implementation, policy recommendations, and practical applications of plant psychedelics, including the variety of Indigenous voices.

This approach, as well as the overarching aims, are similar to the Reciprocity pillar of ARC. The first step of our focus groups was to identify key priorities to support the reciprocity and sustainability of psychedelics, and subsequently translate these into actionable recommendations. These findings are currently being prepared for a separate publication as they go beyond the scope of the present paper which aims to introduce the ARC framework *per se.*

Holding reciprocity as a core value in an ethical framework is hoped to contribute to a culture that makes psychedelic medicines available in a way that respects the lineages of Indigenous knowledges, that are essentially–not accidentally–coupled with many of the psychedelic plants on which Western psychedelic medicines are based (Devenot et al., 2022). Protecting participants and patients as psychedelics move into the mainstream is essential, but equally it is essential to create an environment which supports the autonomy and protection of traditional carriers of these medicines. In this way, the Reciprocity pillar of the ARC framework is interconnected with the Access and Conduct pillars, whereby the values of humanity instilled by an inclusive worldview are incorporated across all pillars.

The issues discussed here are by no means novel or limited to developments in the psychedelic therapy field. The exploitation of natural resources and traditional knowledges relates to much broader concerns than plant medicines. However, with the rapid developments of psychedelic medicines in the West, and the benefits as well as risks that these expansions may bring, there is an opportunity to consider how best to develop this sector in a fair manner, so that benefits are not limited to (largely) Western companies but rather shared with the traditional knowledge bearers who have paved the way for current developments and without whom today’s “psychedelic renaissance” in the Global North might not be happening. These issues go deeper than the commercialization of psychedelic medicines, touching on values of how to treat nature and humanity. It is the responsibility of PAT practitioners, researchers, and other stakeholders to reflect on the ethical dilemmas caused by the commercialization of nature and the sacred, and also to rise to the challenge of developing impactful initiatives toward reciprocity and sustainability.

### 2.3. Conduct

The “conduct” arm of the ARC framework concerns how those involved in developing and delivering psychedelic-assisted therapy carry out their activities, and how PAT is made available to patients and clinical trial participants. Ethical dilemmas occur at every level of the psychedelic therapy system, from the participant-therapist dyad to the conduct of teams, professionals, and the wider socio-political system (Anderson et al., 2020; Read and Papaspyrou, 2021; Thal et al., 2021). Conduct, within the ARC framework, concerns the values and processes involved in developing and delivering PAT, and it is therefore inextricably linked to access and reciprocity. To meaningfully consider the ethics of conduct at every level is a necessarily arduous task, which will take the concerted efforts of many over time.

With the rapid expansion of PAT in clinical trials there is an immediate need to consider the complexity of the participant-therapist dyad in the clinic room (Thal et al., 2021) and therefore this is a key focus. At present, there is no formal certification process for becoming a psychedelic therapist and what (if any) qualifications or training should be required has yet to be established (Phelps, 2017; Williams M. L. et al., 2021). Whilst therapists are governed by the standards of their professional regulating bodies, these ethical codes and practice guidelines do not cover work with psychedelics (Phelps, 2017; Brennan et al., 2021; Thal et al., 2021). Equally, many “psychedelic sitters” both inside and outside research environments are not therapeutically trained and so are not governed by any therapeutic ethical codes. As a result, there are few places offering support or guidance on the complexity of this work. Issues pertaining to the use of therapeutic touch, power dynamics, and boundary transgressions have already begun to surface (McLane et al., 2021).

As patients enter into highly vulnerable and even regressed states with psychedelics, challenging aspects of the ordinary therapeutic relationship and process are amplified (Grof, 2000; Brennan et al., 2021; Read and Papaspyrou, 2021; Thal et al., 2021; Murphy et al., 2022). Viewed through a psychotherapeutic lens, these include complex transferential issues, anxieties, and psychological defenses and enactments; all of which may impede improvement or lead to adverse outcomes if not managed appropriately (Grof, 2000; Thal et al., 2021). Questions such as how to obtain informed consent (Grof, 2000; Smith and Sisti, 2020) how best to support participants who have had spiritual experiences (Grof, 2000), or how to approach the emergence of possible new memories of abuse (Thal et al., 2021) or collective trauma (Williams M. T. et al., 2021) are just a few of the ethical dilemmas practitioners are facing. There are questions as to who this treatment can help and how (Murphy et al., 2022) when it might be unhelpful or harmful (Anderson et al., 2020) and how to work in culturally sensitive ways and with marginalized groups (Anderson et al., 2019; Williams et al., 2020; Williams M. T. et al., 2021).

There is a complex myriad of ethical and clinical issues therapists, sitters and participants can be presented with, which highlights the urgent need for comprehensive ethical and practice guidelines for PAT. Existing documents of this kind (Bevir et al., 2019; Carlin and Scheld, 2019; Guild of Guides, 2020; North Star Ethics Pledge, 2020; Council on Spiritual Practices, 2022; From The Conclave, 2022; Murphy et al., 2022; PAC, 2022) have typically been developed for a specific context and a specific psychedelic substance. We plan to integrate, develop, and expand on these with the involvement of relevant stakeholders such as past participants, therapists of different therapeutic orientations, sitters, and researchers which will be reported in a separate publication. Regardless of theoretical orientation, profession, or context, these guidelines will provide helpful tools for working safely and ethically. These fundamentals of practice can then be used along with others to inform trainings, certification processes, and minimum professional standards. Established standards of care will provide an essential level of professionalism and containment to practitioners supporting the therapeutic process of PAT. It would be a disservice to the depth of this therapeutic modality to suggest simple solutions to the complex questions this work presents. Rather than a set of rigid or inflexible rules, the ARC framework intends to suggest guidelines which are expected to continue to evolve as more is learned and understood about the use of PAT.

## 3. Co-design of the ethics statements

**FIGURE 2:**
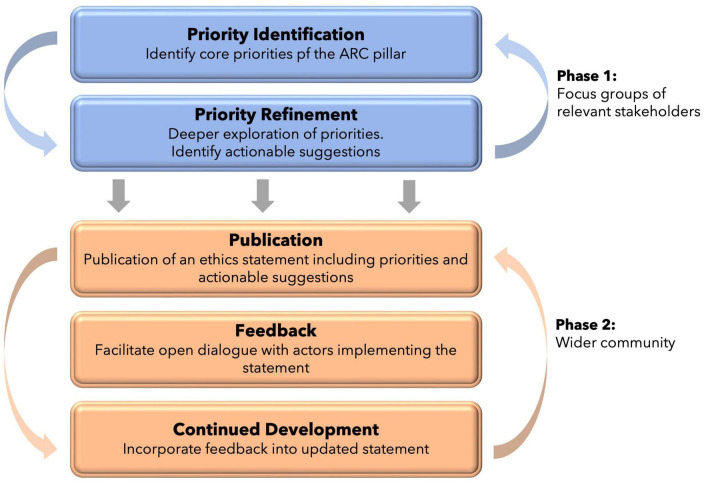
The dual-phase process for co-design of the three ethics statements of ARC. Phase 1 refers to co-design within small focus-groups. Phase 2 refers to co-design with the wider community through implementation and continued feedback.

Co-design is an approach being widely adopted in research, policy, and service design, and refers to the active and deliberate involvement of different stakeholders in exploring, developing and evaluating initiatives (O’Brien and Vincent, 2003; Boyd et al., 2012; Blomkamp, 2018; Bevir et al., 2019; Close et al., 2021; Eseonu, 2022). Not only is co-design a tool for better decision making, but it also considers the influence of existing power dynamics and inequalities, and gives stakeholders an opportunity to address fractionization (Bevir et al., 2019). Here, we employ co-design in two ways (1) the use of focus groups in the generation of the initial ethical statements, and (2) opening up for feedback from the wider transdisciplinary communities of stakeholders involved in PAT, based on real-world practical implementation (Figure 2).

At the time of writing, the ethical statements for the three ARC pillars are within the first stage of development: the use of focus groups to develop the ethics statements. Given the complexity of each theme, a statement for each of the three pillars will be developed independently. The process is iterative, whereby key ethical principles are identified, explored, and molded through multiple open discussions with a focus group of different stakeholders. The stakeholders have been identified to be representative of the actors most closely implicated in the ARC pillar in question, and come from research, industry, community, anthropological, policy, and indigenous contexts. The goal of these focus groups is not to produce the *answers* but to bring important questions and potential solutions into our shared awareness, so as to promote future focus, expansion, and research on important themes. As the coordinators of ARC, our role is to elicit the guiding principles, priorities, and wider substance of the ethical framework. Each pillar is working under an independent timeline, however, work began on this process in August 2021.

At the end of this phase of the process, a statement on ethics and practice will be produced and published for each of the three pillars. Each statement will provide recommendations, guidelines and thinking tools to provide pragmatic steps others can incorporate into their practice in their own context. Importantly, we do not view this as the end of the process. Once publicly available, we invite further feedback from the wider psychedelic community on the implementation of the framework. We envision that all three statements will hold relevance for most PAT clinical contexts and holding them all under the shared ARC framework allows for a more coherent integration with one another. It is hoped that this process will empower psychedelic research, practitioners and organizations to tackle these ethical issues within the scope of their own work.

## 4. Conclusion

Meeting the unique and multifaceted ethical and practice demands of PAT will take careful forethought, interdisciplinary collaboration, and humility. In line with the current developments in psychedelic medicine, and the nascent psychedelic industry, the incorporation of guidelines addressing safety, reciprocity and equity is vital yet difficult to develop and challenging to implement. The future potential of PAT can only be fully realized if the broader socio-cultural context is considered, and both patients and traditional communities are included as key stakeholders, and decision makers.

ARC represents a framework that embodies reciprocity, protects conduct, and prioritizes equity of access, valuing the knowledge and experience of traditional Indigenous healers, therapists, scientists, participants and the wider community. It is hoped that this will springboard important ethical conversations in research, therapy and public domains, so that the safe and ethical development of these treatments can be at the forefront when the field of PAT matures.

## Data availability statement

The original contributions presented in this study are included in the article/supplementary material, further inquiries can be directed to the corresponding author.

## Author contributions

MS, AM-B, and AS conceived the study. MS had oversight of the writing of the manuscript with contributions from AM-B, AS, JB, HT, and RM. All authors developed the study and revised the manuscript.

## Footnotes

1 Many of the topics discussed herein are relevant both to *classic* psychedelics (i.e., psilocybin, LSD, DMT), and psychedelic-like compounds such as MDMA and ketamine. We therefore take “PAT” to encompass psycho-pharmacotherapy approaches using all the above compounds.

2 We use the term “marginalized” for groups whose needs have are precluded from mainstream social, educational, economic and cultural life (Hynie, 2018), and/or have been historically subjugated in research and medicine. This includes (but is not limited to), groups marginalized due to their racial or ethnic group, immigration status, gender or sexuality, economic deprivation or socio-economic status, and all other characteristics recognized by the United Kingdom’s Equalities Act 2010 (Cookson et al., 2021).

3 The case of Maria Sabena provides a well documented example, in which the Western use of sacred plant medicines proved devastating for the traditional healer (Nichols, 2020; Gerber et al., 2021).
